# Histological, immunohistochemical and mRNA gene expression responses in coeliac disease patients challenged with gluten using PAXgene fixed paraffin-embedded duodenal biopsies

**DOI:** 10.1186/s12876-019-1089-7

**Published:** 2019-11-15

**Authors:** Juha Taavela, Keijo Viiri, Alina Popp, Mikko Oittinen, Valeriia Dotsenko, Markku Peräaho, Synnöve Staff, Jani Sarin, Francisco Leon, Markku Mäki, Jorma Isola

**Affiliations:** 1 Department of Paediatrics, Tampere Centre for Child Health Research, Faculty of Medicine and Health Technology, Tampere University, Tampere University Hospital, Tampere, Finland; 2 Department of Internal Medicine, Central Finland Central Hospital, Jyväskylä, Finland; 3 University of Medicine and Pharmacy “Carol Davila” and National Institute for Mother and Child Health “Alessandrescu-Rusescu”, Bucharest, Romania; 4 Department of Gynaecology and Obstetrics, Tampere University Hospital, Tampere, Finland; 5 Laboratory of Cancer Biology, Faculty of Medicine and Health Technology, Tampere University, Tampere, Finland; 6 Jilab Inc., Tampere, Finland; 7 Celimmune LLC, Bethesda, MD USA

**Keywords:** Coeliac disease, Biopsy, Morphometry, Immunohistochemistry, mRNA, PAXgene, Digital histopathology, RNA, Histology, Gluten

## Abstract

**Background:**

There is an unmet need for novel treatments, such as drugs or vaccines, adjunctive to or replacing a burdensome life-long gluten-free diet for coeliac disease. The gold standard for successful treatment is a healed small intestinal mucosa, and therefore, the outcome measures in proof-of-concept studies should be based on evaluation of small intestine biopsies. We here evaluated morphometric, immunohistochemical and messenger RNA (mRNA) expression changes in coeliac disease patients challenged with gluten using PAXgene fixed paraffin-embedded biopsies.

**Methods:**

Fifteen coeliac disease patients were challenged with 4 g of gluten per day for 10 weeks and 24 non-coeliac patients served as disease controls. A wide array of histological and immunohistochemical staining and mRNA-based gene expression tests (RT-qPCR and RNAseq) were carried out.

**Results:**

Digital quantitative villous height: crypt depth ratio (VH: CrD) measurements revealed significant duodenal mucosal deterioration in all coeliac disease patients on gluten challenge. In contrast, the Marsh-Oberhuber class worsened in only 80% of coeliac patients. Measuring the intraepithelial CD3^+^ T-lymphocyte and lamina propria CD138^+^ plasma cell densities simultaneously proved to be a meaningful new measure of inflammation. Stainings for γδ T cells and IgA deposits, where previously frozen samples have been needed, were successful in PAXgene fixed paraffin-embedded samples. Messenger RNA extraction from the same paraffin-embedded biopsy block was successful and allowed large-scale qRT-PCR and RNAseq analyses for gene expression. Molecular morphometry, using the mRNA expression ratio of villous epithelium-specific gene APOA4 to crypt proliferation gene Ki67, showed a similar significant distinction between paired baseline and post-gluten challenge biopsies as quantitative histomorphometry.

**Conclusion:**

Rigorous digitally measured histologic and molecular markers suitable for gluten challenge studies can be obtained from a single paraffin-embedded biopsy specimen. Molecular morphometry seems to be a promising new tool that can be used in situations where assessing duodenal mucosal health is of paramount importance. In addition, the diagnostically valuable IgA deposits were now stained in paraffin-embedded specimens making them more accessible in routine clinics.

## Background

Coeliac disease is an autoimmune disorder in which dietary gluten causes a gradually developing villous atrophy and crypt hyperplasia in small intestine mucosa [1]. Patients may present with severe gastrointestinal symptoms, extraintestinal manifestations such as dermatitis herpetiformis, or be asymptomatic but diagnosed by at-risk group screening [2]. Currently, the only therapeutic option is a life-long, strict gluten-free diet, which is burdensome and limits the normal day-to-day life [3]. Gluten is abundant in everyday diets, and gluten contamination of otherwise gluten-free foods is difficult to avoid [4]. In fact, 20–50% of treated coeliac patients report gastrointestinal symptoms [5]. Hence, coeliac patients have expressed a desire to use novel drugs or vaccines as adjunctive or even alternative treatments for coeliac disease [6]. Mucosal healing is the ultimate goal in coeliac disease dietary treatment, but this is often not achieved, as reviewed by Ilus et al. [7].

Gluten challenge studies are a rising entity for undergoing and upcoming drug and vaccine trials for coeliac disease [8]. Normal food contains approximately 10–20 g of gluten per day, and a daily dose of only 1–3 grams [9, 10] or even 50 micrograms [11] of gluten can induce measurable histological changes in gluten challenge studies. The mucosal damage depends on the dose and duration of the gluten challenge [9, 10]. Patient-related outcomes (PROs) can also be used as a disease severity indicator [12]; however, the symptoms of an individual patient may not reflect the mucosal status during the relatively short gluten challenge [13, 14]. Hence, histological analyses have been used and are considered necessary in clinical phase II proof-of-concept drug trials to demonstrate objective morphological and inflammatory gluten-induced change in coeliac disease patients [15].

In gluten challenge studies, the technical quality of the histological biopsy samples must be rigorously monitored and ensured to produce reliable results, as there are several pitfalls in obtaining the biopsy, orientating the biopsy section and evaluating the section [16–18]. Conventionally, duodenal biopsies are fixed with formalin and embedded in paraffin (FFPE), which allow the analysis of duodenal morphology, density of intraepithelial lymphocytes (IELs) and proteins through label-free quantitative mass spectrometry [19], but many immunohistochemical markers and messenger RNAs (mRNAs) that would provide detailed molecular data cannot be analysed from the same biopsy specimens when formalin is used as a fixative. Similarly, samples stored in RNA-preserving reagents, such as RNAlater, cannot be used for histology or immunohistochemistry [20]. Frozen sections can be used for both, but rapid deep-freezing and transportation of the biopsies is difficult to arrange in multicentre clinical drug trials. Moreover, high-quality frozen sectioning of mucosal biopsies is technically challenging. The new PAXgene molecular fixative allows histological, immunohistochemical, and mRNA studies [21, 22], and thus, it may enable a much wider array of histological and biological outcome measures from single duodenal biopsies that would give important information, especially in the new coeliac disease drug trials. Hence, we studied here quantitative histological measurements, multiple immunohistochemical markers and RNA markers with PAXgene fixative in a gluten challenge setting in coeliac disease patients.

## Methods

### Patients and biopsies

Altogether, 15 adult patients with previously diagnosed coeliac disease were recruited for a gluten challenge study. Coeliac disease diagnosis was ensured in all coeliac disease patients from patient records before gluten challenge. This comprised positive coeliac disease autoantibody tests and the characteristic histological changes of villous atrophy and crypt hyperplasia in the duodenal biopsy. All celiac disease patients had been on a gluten-free diet for at least 1 year. The patients were subjected to a gluten challenge of 4 g of gluten per day. Biopsies were taken before the gluten challenge, and the same study subjects were biopsied again 10 weeks after the beginning of the gluten challenge. Twenty-four non-coeliac disease control patients undergoing clinically indicated upper gastrointestinal endoscopies were invited to participate. For these patients, endoscopy was performed due to unexplained abdominal symptoms, dyspepsia or symptoms of gastroesophageal reflux without suspicion of coeliac disease. Coeliac disease was excluded in the control group by normal histopathology findings in routine pathologic examination of duodenal biopsies. They were also negative for serum coeliac disease autoantibodies.

Six biopsy samples were taken from the distal duodenum and placed in a multi-compartment tissue cassette, which was immersed in PAXgene fixative for 1–4 h and transferred to the proprietary storage solution in PAXgene dual-chamber fixative containers (Qiagen #765112, Venlo, Netherlands). Biopsies were stored at + 4 °C and transferred to the central pathology laboratory at ambient temperature. Of the 24 non-coeliac patients, 15 patients’ biopsies were fixed with routine formalin fixative, five with PAXgene fixative and four subjects had both formalin and PAXgene fixed specimens.

### Biopsy processing and staining

PAXgene-fixed samples were processed for paraffin block embedding (PaxFPE) using a standard formalin-free paraffin-infiltration protocol. Each biopsy was embedded in a separate paraffin block under a dissection microscope and aimed for a cutting plane perpendicular to the mucosal lumen surface to orientate the specimens correctly [16]. To measure villous height (VH), crypt depth (CrD), and their ratio (VH: CrD), the slides were stained with haematoxylin and eosin.

Immunohistochemical staining was carried out, and lymphocytes were stained separately for CD3, CD4, CD8, CD19, CD138, CD163, FOXP3, Ki67, CyclinB1 and γδ. Additionally, double immunofluorescence of CD3/CD8 lymphocytes and IgA deposits, i.e., visualizing duodenal mucosal IgA targeting extracellular TG2, was carried out [23]. The staining procedures are described in detail in Additional file 1.

### Digital histomorphometry

**Fig. 1:**
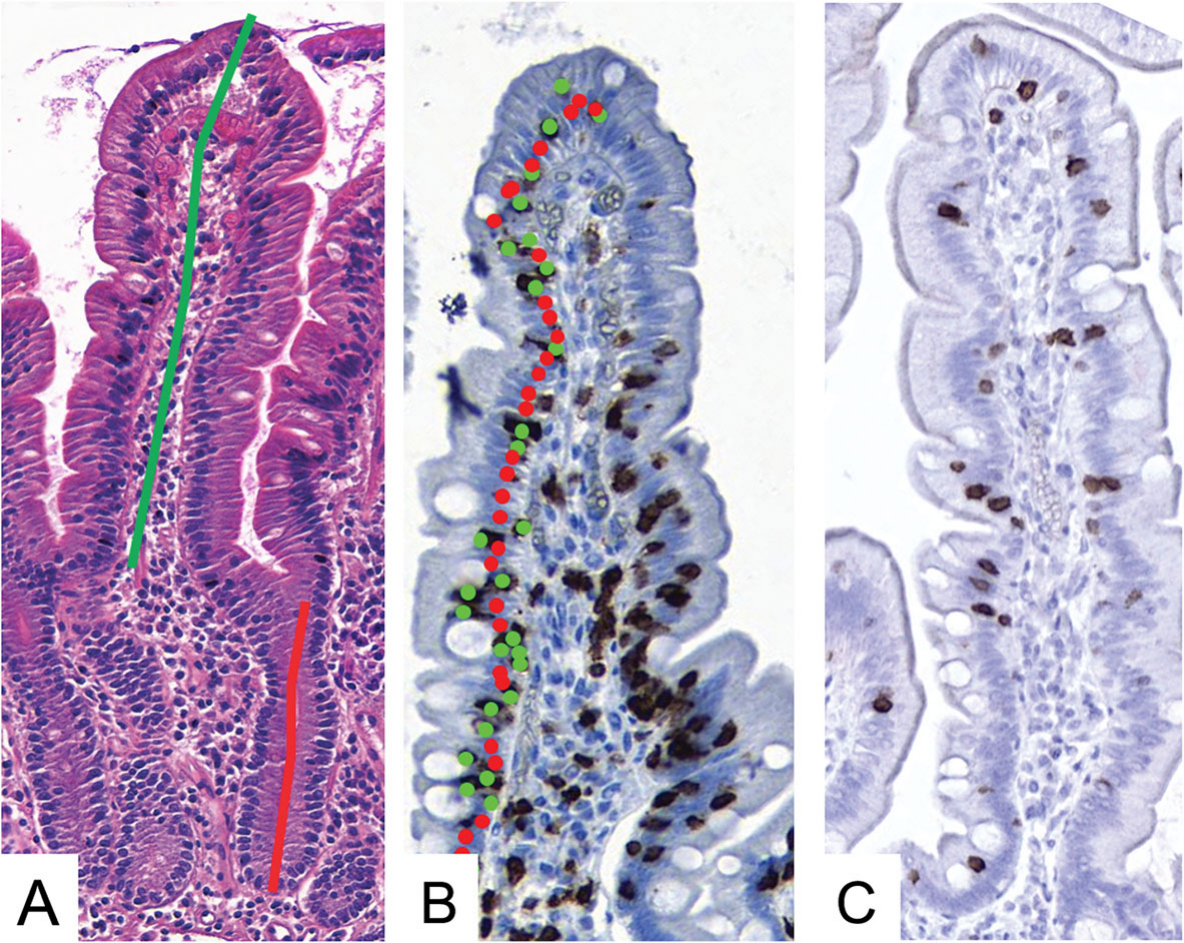
Examples of digital measuring in coeliac disease biopsy specimens. Villous height, crypt depth and villous height: crypt depth ratio measurements in haematoxylin and eosin–stained specimens (**a**), CD3^+^ intraepithelial lymphocyte density count per enterocyte (**b**) and γδ^+^ intraepithelial lymphocyte staining in PAXgene-fixed specimens (**c**). Measurements were performed with Celiac Slide Viewer

All slides were scanned as whole-slide images using a SlideStrider scanner at a resolution of 0.28 μm per pixel (Jilab Inc., Tampere, Finland). Images were stored as JPEG2000 files in the image server and viewed over the internet with web-based client software developed for this study (Celiac Slide Viewer). The sections were analysed according to our standard operating procedure [16]. The small intestinal mucosal VH and CrD were evaluated from at least three separate villous-crypt units, and the result was given as the average of the ratios. VH and CrD were measured digitally by drawing polylines (Fig. 1a). Two academic observers (JT, AP) analysed all slides independently and were unaware of the clinical data or laboratory findings of the patients. A crucial step in the procedure was that an experienced evaluator, besides producing results with acceptable interobserver and intraobserver morphometric variation, identified cases that had inadequate sample material and/or poor biopsy orientation, where measurements of villous-crypt units are not reliable [16]. In the case of poor orientation that resulted in tangential cuttings, the evaluator asked for recuttings until reliable morphological readouts could be obtained.

CD3^+^ and γδ^+^ IELs were measured within the epithelium of at least 300 enterocytes using the Auto-IEL tool of Celiac Slide Viewer (Fig. 1b and c), and the result was given as IEL density expressed per 100 enterocytes (ECs). In IEL measurements, the results are independent of biopsy orientation, and re-cutting of specimens is not needed [16]. CD138^+^ plasma cells in the lamina propria were enumerated automatically and adjusted to the measurement area with ImmunoRatio2 software, which is part of the Celiac Slide Viewer.

### Messenger RNA analyses

Total RNA was extracted from PAXgene-fixed and formalin-fixed specimens (30 to 50 unmounted tissue sections of a single biopsy) according to the manufacturer’s instructions. Quantitative real-time PCR using RT [2] Profiler PCR arrays and RNAseq were carried out and the data were analysed. Methods are described in detail in the Additional file 1.

### Statistics

Quantitative data are expressed as the means, percentages and ranges. The two-tailed pairwise Mann-Whitney U test and the Kruskal-Wallis test were used to compare the differences between the groups. All statistical testing was performed using Predictive Analytics Software Statistics (PASW) version 18 (IBM, USA).

## Results

### Mucosal architecture assessments

**Fig. 2:**
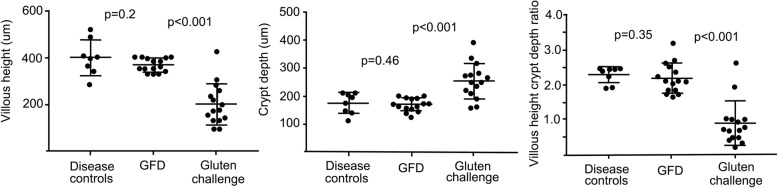
Mucosal morphology in gluten challenge. The measurement of villous height, crypt depth and villous height: crypt depth ratio in non-coeliac disease control patients, in coeliac disease patients on a gluten-free diet (GFD) before gluten challenge and then in coeliac disease patients after the gluten challenge. There was no significant change in non-coeliac disease control patients or coeliac disease on a gluten-free diet in any parameter, but as expected, significant changes were seen between the samples taken before and after the gluten challenge in coeliac disease patients

**Fig. 3:**
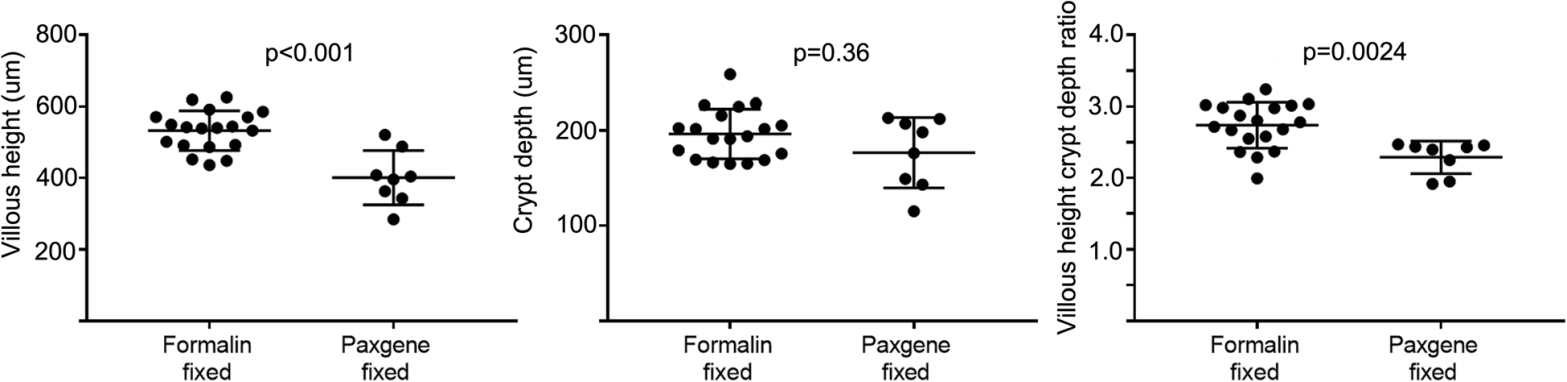
Comparison of mucosal morphology between formalin and PAXgene fixed specimens. Villous height, crypt depth and villous height:crypt depth ratio measurements in routine formalin-fixed biopsies and in PAXgene-fixed biopsies. The villi were significantly lower in PAXgene-fixed samples, but crypts were unaffected. There was also a trend towards lower villous height:crypt depth ratio in PAXgene-fixed samples

VH, CrD and their ratio were similar between disease control patients and coeliac disease patients on a gluten-free diet (Fig. 2). In coeliac disease patients, a significant change was seen between baseline and post gluten challenge values in PaxFPE samples (*p* < 0.001, Fig. 2). The PaxFPE samples of non-coeliac disease controls were readable in eight out of nine samples, and the median VH, CrD and VH: CrD were 401 μm (284–521), 177 μm (range 115–213) and 2.3 (range 1.9–2.5), respectively; in coeliac disease patients on GFD, the values were 369 (range 327–406), 172 (range 126–203) and 2.2 (range 1.7–3.2; *n* = 15/15); and after the gluten challenge, they were 202 μm (range 96–425), 255 μm (range 158–392) and 0.9 (range 0.2–2.6; n = 15/15). In FFPE samples of non-coeliac disease control patients, the VH, CrD and VH: CrD were 533 μm (range 448–625), 196 μm (165–258) and 2.7 (range 2.0–3.2; *n* = 19/19), respectively, showing significantly higher VH (*p* < 0.001) and VH: CrD (*p* = 0.0024) than in PaxFPE (Fig. 3). In pairwise analysis (*n* = 4) of FFPE and PaxFPE specimens, the median VH, CrD and VH: CrD were 518 μm (448–591), 200 μm (164–258) and 2.6 (2.3–2.8), respectively; in PaxFPE the values were 401 μm (343–488), 176 μm (143–212) and 2.3 (1.9–2.5).

### Mucosal T- and B-lymphocytes

**Fig. 4:**
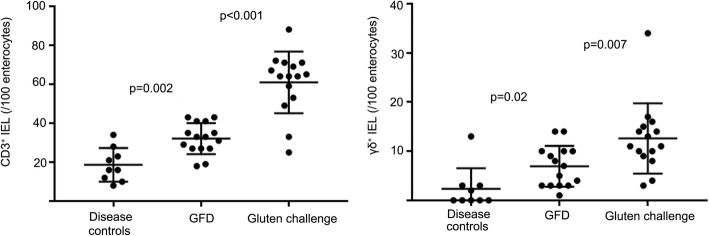
Mucosal inflammation in gluten challenge. CD3^+^ and γδ^+^ intraepithelial lymphocyte (IEL) density in non-coeliac disease control patients, in coeliac disease patients on a gluten-free diet (GFD) and in coeliac disease patients after gluten challenge

Intraepithelial CD3^+^ and γδ^+^ IEL densities were significantly elevated (p = 0.002; *p* = 0.02) in the coeliac disease patients on GFD with a mean of 32.1 per 100 ECs (range 18–43) and 6.9 per 100 ECs (range 1–14) compared to non-coeliac disease control patients 18.7 per 100 ECs (range 8–34) and 2.3 per 100 ECs (range 0–13), respectively. In the gluten challenge, the CD3^+^ and γδ^+^ IEL densities increased to a mean of 60.9 per 100 ECs (range 49–88) and a mean of 12.6 per 100 ECs (range 3–34; Fig. 4) and were significant (*p* < 0.001, *p* = 0.007).

**Fig. 5:**
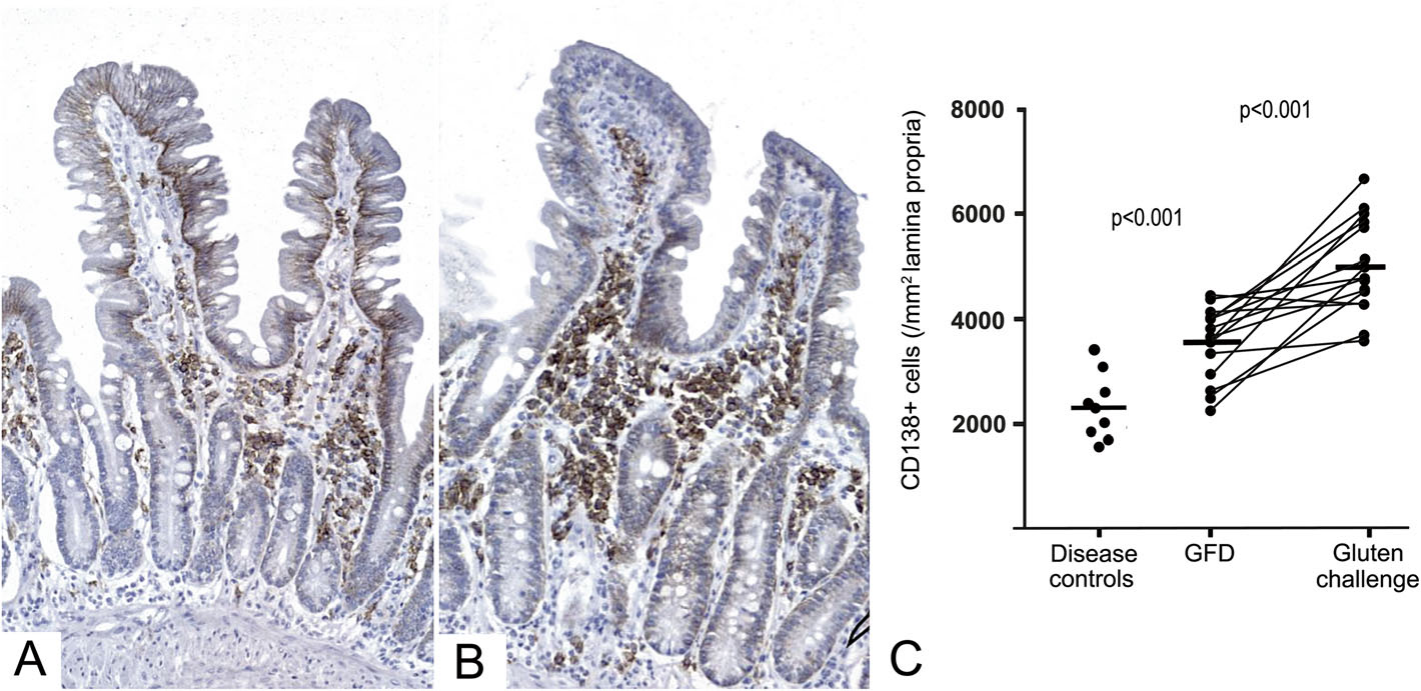
CD138^+^ lymphocytes in gluten challenge. CD138^+^ lymphocytes in lamina propria stained from PAXgene-fixed duodenal biopsies before (**a**) and after the gluten challenge (**b**) in a coeliac disease patient. The CD138^+^ lymphocyte density was increased in coeliac disease patients on gluten-free diet (GFD) compared with non-coeliac disease control patients; the CD138^+^ lymphocyte density also increased significantly after the gluten challenge in coeliac disease patients (**c**)

To explore the role of B-cell lineage, we stained the study material for CD138, which specifically showed plasma cells in the lamina propria (Fig. 5). The CD138^+^ lymphocytes were elevated significantly in the coeliac disease patients on GFD, with a mean of 3580 per mm^2^ of lamina propria (range 2320–4450), compared to disease control patients, who had a mean of 2390 per mm^2^ (p < 0.001; range 1650–3140). After gluten challenge, a significant increase to a mean of 5013 per mm^2^ of lamina propria was observed (p < 0.001; range 3709–6108).

### Conversion of villous height and crypt depth measurements to Marsh-Oberhuber classification

Conversion from the continuous injury parameters VH: CrD and IEL to the grouped Marsh-Oberhuber injury classification grading system (classes 0, 1, 2, 3a, 3b, 3c) can be achieved using the conversion table provided by Adelmann et al. [24] However, based on the observed shrinkage of villi in PAXgene fixative and the consequent lower VH: CrD values in PaxFPE samples, we re-formulated the conversion table to match the results obtained with PaxFPE biopsies (Additional file 2: Table S1). With this conversion table, we observed a clinically significant Marsh score change (2 or more classes) in 80% of cases after gluten challenge (Table 1) [25]. For comparison, when using the continuous injury variable VH: CrD ratio as the outcome, clinically significant worsening (0.4 units or more) [16] was evident in all coeliac disease patients.

**Table 1:** The change in duodenum estimated before and after the gluten challenge with the Marsh-Oberhuber classification.change in duodenum estimated before and after the gluten challenge with the Marsh-Oberhuber classification

Marsh class change	Number of study subjects (%)
No change	1 (7%)
Change by one class^a^	2 (13%)
Change by two classes^b^	8 (53%)
Change by three classes^c^	4 (27%)

^a^From M1 to M2, M2 to M3a, M3a to M3b, or M3b to M3c

^b^From M1 to M3a, M3a to M3c, or M2 to M3b

^c^From M1 to M3b, M2 to M3c, or M1 to M3c

### Immunofluorescence assays

The staining of IgA deposits using double immunofluorescence staining of IgA and transglutaminase 2 (Additional file 3: Figure S1) was successful with PAXgene, which so far has been possible only on frozen sections. Also, the staining reactions were strong in dual-colour indirect immunofluorescence staining of CD3^+^CD8^−^ IELs (Additional file 3: Figure S1) and in an immunohistochemical panel (CD4, CD8, CD19, CD163, and FOXP3) consisting of markers known to be relevant for the pathogenesis of coeliac disease (Additional file 4: Figure S2). Formalin fixation provides the same staining possibilities as this immunohistochemical panel but lacks the possibility to stain IgA deposits and γδ^+^ T cells.

### Gene expression profiling

The main technical advantage of the PaxFPE technology is the possibility to extract RNA for molecular gene expression studies. For this, we extracted RNA from the same paraffin blocks that were first used for histological sections (see Additional file 1). Despite the small amount of tissue used for RNA extraction, 3 to 14 micrograms RNA was obtained. The quality of RNA, as estimated by the RQN values, varied from 2.1 to 5.6, which is typical for the RNA obtained from intestinal tissues. Somewhat compromised RQN values notwithstanding, we were able to perform gene expression analyses. To exemplify this, we analysed the mRNA levels of two genes whose expression is correlated with coeliac disease mucosal damage, namely, APOA4 [26] and Ki67 [27], by RT-qPCR and RNAseq.

For comparison, we tested the possibility of RNA analysis also with traditional formalin-fixed small bowel biopsies from three patients (see Additional file 1). The RNA yield from these samples was very low ranging from 0.092 to 0.12 micrograms. Understandably, RQN values could not be defined and RT-qPCR analyses did not give any signal in these specimens. This finding is in agreement with previous studies [20].

In RNAseq, the total number of reads was 196,558,237, and there were 4,820,392 reads on average per sample. The median of detected genes across all 36 samples was 19,863 (standard deviation 1910). The mean reads per unique molecular identifiers (UMI) was 4.12 (standard deviation 0.36). In 89% of samples, the number of detected genes was over 17,000. The reads per UMI was over 3 in 97% of samples.

**Fig. 6:**
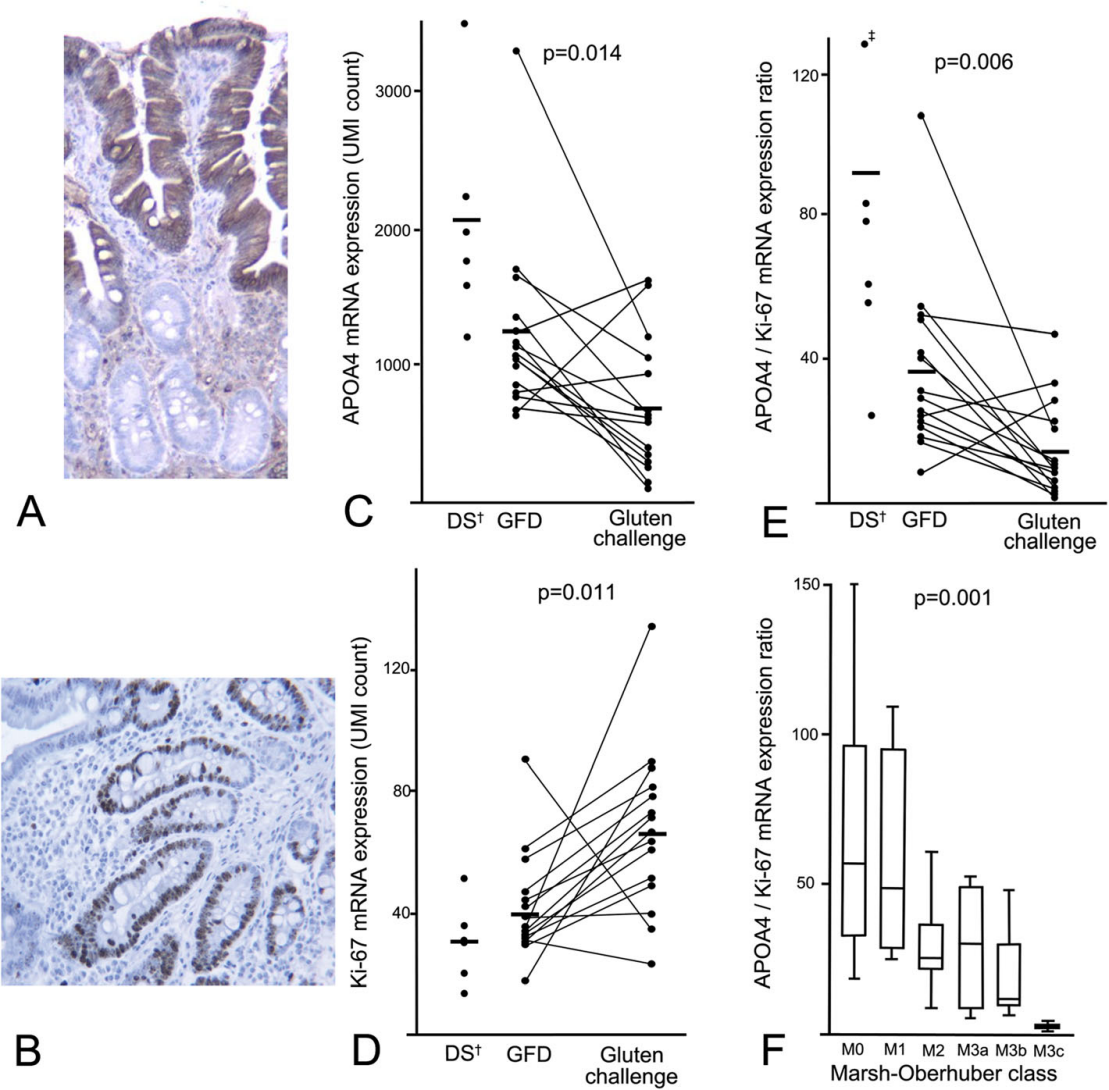
Molecular morphometry in gluten challenge. Immunohistochemical stainings of APOA4 (**a**) and Ki67 (**b**) in PAXgene fixative and the mRNA levels of these genes in disease controls (DS) and before and after the gluten challenge. In pairwise analysis of each patient, the expression of the villous epithelium gene APOA4 decreased significantly after gluten challenge (**c**), whereas crypt cell proliferation, as indicated by Ki67 messenger RNA, increased significantly (**d**). The ratio of these two genes appeared to be the most prominent marker (**e**). APOA4 and Ki67 ratio and Marsh-Oberhuber classification, which was obtained via the conversion table (presented in Additional file 2: Table S1) from the villous height:crypt depth ratio and intraepithelial lymphocyte count, had a significant correlation, thus displaying the clinical link between these mRNA markers and mucosal injury (**f**). †Disease controls, DS; ‡ one sample was out of the measurement scale, with a value of 257

Figure 6a and b show the villous epithelial staining of APOA4 and the crypt epithelial staining of Ki67. Figure 6c and d show the RNAseq-extracted mRNA levels of APOA4 and Ki67 in disease controls and in coeliac disease patients before and after gluten challenge, as individual plots and group means. The ratio of APOA4 to Ki67 encompasses both the mRNA levels of villi (APOA4) and crypts (Ki67) (Fig. 6e). Hence, these results present the gluten-induced changes that occur in molecular VH (APO4), CrD (Ki67) and VH: CrD (APOA4/Ki67) alongside the architectural mucosal changes in coeliac disease. Figure 6f further demonstrates the changes between the APOA4/Ki67 mRNA ratio and the histological Marsh-Oberhuber classes of biopsy specimens. Additionally, the correlation coefficient between the APOA4:Ki67 mRNA ratio and the morphometrical VH: CrD was good, at 0.634 (*p* < 0.001), and APOA4:Ki67 mRNA ratio correlated well with the IEL density (− 0.575, p < 0.001). The correlation coefficient of APOA4 between the two methods, RT-PCR and RNAseq, was excellent (0.95), indicating that the RNA analyses are highly reproducible.

## Discussion

In coeliac disease, the mucosal architectural injury and intra-epithelial inflammation induced by the ingested gluten are both biological continuums, which can be measured morphometrically [9, 16]. However, in the clinic, the status of mucosa is most often depicted with categorical classifications, such as the Marsh-Oberhuber classification, where the injury continuum is grouped in a non-biological way into 6 classes [28]. This is valid when diagnosing coeliac disease, but for gluten challenge studies, which are used in drug or vaccine trials, more precise measurements are needed. In gluten challenges, small but significant mucosal changes must be observed in well-treated coeliac disease patients who have ingested low or moderate amounts of gluten for a short period of time. For this purpose, quantitative morphometric measurements such as mucosal architectural changes (VH: CrD) and inflammation (IEL density) are essential [15]. Indeed, clinically significant mucosal changes are seen within one single Marsh-Oberhuber class [10, 16]. In this study, a conversion table was created in which duodenal mucosal morphometric measurements could be changed into Marsh-Oberhuber classes in PAXgene-fixed samples. It was evident with this conversion table that categorical classification would have missed 20% of patients showing evident mucosal change in VH: CrD measurements. This finding agrees with previous studies showing poor performance of categorical classifications in gluten challenge studies [9, 11, 16]. Measurement of IELs provides another continuous, quantifiable parameter that is not fully considered in any of the categorical classifications despite its importance as an early marker of inflammation in short gluten challenges [9].

Frozen specimens have been used to stain the γδ ^+^ IELs and IgA deposits, which have been used widely as markers for latent, i.e., early-onset, coeliac disease [23, 29–32]. The rapid freezing of duodenal biopsies makes it difficult to arrange them such that they are assessable in remote clinical centres, and the transportation of deep-frozen specimens is burdensome as it requires special packaging and shipping. Intriguingly, γδ^+^ IELs and IgA deposit stainings were reproduced with PaxFPE biopsy blocks but not in formalin-fixed samples. This offers a significant new option for clinical routine and potentially for forthcoming coeliac drug trials, as the need for biopsy freezing has been the main hindrance to the wider use of IgA deposit studies in coeliac disease despite its almost 100% accuracy [32].

Another promising marker for measuring coeliac disease outcome is quantitation of the antibody-producing plasma cells in the lamina propria [33]. Interestingly, in a new article by Høydahl et al. the plasma cells are suggested to be the most prevalent antigen presenting cell in celiac disease and a potential target for treatment [34]. In our study, the number of CD138^+^ plasma cells was somewhat elevated in coeliac disease patients on a strict gluten-free diet, but it increased highly significantly upon a moderate and short-term gluten challenge. With digital image analysis, it was feasible to measure the number of CD138^+^ cells per mm^2^ of lamina propria, thereby providing an observer-independent measure of gluten-dependent coeliac disease activity. Measuring IELs and lamina propria B cell density responses simultaneously could constitute a new duodenal inflammation outcome marker in gluten challenge studies. It remains to be seen whether quantitation of TG2-producing plasma cells will give added value in short-term gluten challenge studies [35].

There are several pitfalls in the histological measurement of biopsies [16, 17]. The agreement between local and central pathologist in Marsh-Oberhuber classes is only 42%, and even the diagnoses differed (normal vs coeliac disease) in 7% of patients in a large European multicentre study [18]. Thus, objective quantitative measurements using digital histomorphometric tools and new immunohistochemistry and/or mRNA analyses could provide significant help to assess the degree of damage in the small bowel mucosa. In our mRNA analyses, we found that sufficient material could be harvested from PAXgene-fixed biopsy blocks. Additionally, PAXgene requires less biopsy material than the combination of routine formalin samples and RNAlater assessments. This will make endoscopy easier and faster and, in addition, is a simpler method to store and ship the samples as only one biopsy is needed to acquire both histology and mRNA results. Separate biopsy specimens may also have considerable variation in mucosal morphology and inflammation for which the single specimen analysis provides consistency and removes any biopsy-based discrepancy in results. As an example of the potential use of molecular morphology in duodenal biopsies, we selected the mRNA of a known villous gene marker, APOA4, and a crypt proliferation marker, Ki67, for further study. These markers, and especially the villous:crypt ratio of these two parameters, APOA4/Ki67, appeared to measure coeliac disease activity well in the gluten challenge. APOA4 and Ki67 ratio had a high correlation to mucosal VH: CrD and performed well in the comparison to Marsh-Oberhuber classes (Fig. 6f). Therefore, the use of RNAseq data could be especially valuable to ensure more reliable results when used in combination with routine morphological biopsy assessment in gluten challenge settings. A more detailed bioinformatics-based analysis of the mRNA levels of the ~ 19,000 genes analysed in one RNAseq run will be reported elsewhere (Viiri et al., manuscript in preparation).

## Conclusions

Significant gluten-induced morphological (VH: CrD) and inflammatory (CD3+ IELs and CD138+ lamina propria plasma cells) changes are measurable in gluten challenge studies using digitally measured quantitative variables. With PAXgene fixative it is also possible to acquire a wide array of immunohistochemical stainings and quantitative analyses of mRNA from single paraffin-embedded biopsy specimens. In particular, the possibility to stain IgA deposit and γδ^+^ T cells in paraffin-embedded specimens is a new option for clinicians. The mRNA analyses from duodenal biopsies could serve as a molecular surrogate for the morphometrical villous: crypt ratio in the future.

## Supplementary information

**Additional file 1:** Histological staining procedures, RNA extraction, quantitative real-time PCR, RNAseq and data analysis methods described in detail.

**Additional file 2: Table S1.** Conversion of villous height:crypt depth ratio and CD3^+^ intraepithelial lymphocyte density results of PAXgene-fixed duodenal biopsies to Marsh class and vice versa.

**Additional file 3: Figure S1**. Immunohistochemical stainings in PAXgene fixed specimens. CD3 (A) and CD8 staining (B) of PAXgene-fixed specimens for analysis of CD3^+^CD8^−^ lymphocytes in the diagnosis of refractory coeliac disease. Small intestine mucosal immunoglobulin (Ig) A deposits in duodenal specimens in a coeliac disease patient are also shown. First was normal haematoxylin and eosin staining (C), followed by IgA staining (green (D)) and transglutaminase 2 (TG2) (red (E)). The subepithelial colocalization of IgA and TG2 can be seen in yellow (F).

**Additional file 4: Figure S2**. CD4- (A), CD8- (B), CD163- (C) and FOXP3-stained (D) lymphocytes in PAXgene-fixed specimens.

## Data Availability

The datasets during and/or analysed during the current study are available from the corresponding author on reasonable request.

## Abbreviations

EC: Enterocytes
FFPE: Formalin- fixed paraffin- embedded
GFD: Gluten-free diet
IEL: Intraepithelial lymphocyte
mRNA: messenger RNA
PaxFPE: PAXgene- fixed paraffin- embedded
PRO: Patient related outcome
VH:CrD: Villous height crypt depth ratio
