# Supplementary material for: Histological, immunohistochemical and mRNA gene expression responses in coeliac disease patients challenged with gluten using PAXgene fixed paraffin-embedded duodenal biopsies
Source: BMC Gastroenterol. 2019 Nov 15;19:189. doi: 10.1186/s12876-019-1089-7 (PMC6858741; doi:10.1186/s12876-019-1089-7)
Supplement: Supplementary file 1 — Additional file 1: Histological staining procedures, RNA extraction, quantitative real-time PCR, RNAseq and data analysis methods described in detail. [file 12876_2019_1089_MOESM1_ESM.docx]

**Immunohistochemistry**

Immunohistochemistry was performed on adjacent sections using a standard protocol, consisting of de-paraffinization, heat-induced antigen retrieval (incubation at 121°C for 2 min in 0.01 Tris-EDTA buffer, pH 9.0), blocking endogenous peroxidase (3% H_2_O_2_ for 5 min at RT), and primary antibody incubation (60 min at RT) followed by anti-mouse/anti-rabbit peroxidase polymer and DAB chromogen for detection (HistoFine kit, Nichirei Biosciences, Japan) (Nichirei). Slides were counterstained with haematoxylin and mounted with DPX (Sigma-Aldrich, Missouri, USA). Immunohistochemical staining was carried out with an automated IHC-staining device (Labvision Autostainer; Thermo Fisher Scientific, Massachusetts, USA). The following primary antibody clones and dilutions were used: CD3 (clone SP7, 1:300, Thermo Fisher Scientific), CD4 (clone EP204 1:200, CellMarque (Merck), California, USA), CD8 (clone C8/144B, 1:50, Thermo Fisher Scientific), CD19 (clone LE-CD19, 1:200, Dako (Agilent), California, USA), CD138 (clone EP201, 1:2000), CD163 (clone MRQ-26, 1:100, Dako), FOXP3 (clone SP97, Thermo Fisher Scientific, 1:100), γδ-TCR (clone gamma 3.20, 1:100, Thermo Fisher Scientific), Ki67 (clone MIB-1, 1:2000, Dako), and Cyclin B1 (clone RBT-B1, BioSB, California, USA).

Double immunofluorescence for CD3 and CD8 or for IgA and transglutaminase 2 was carried out using the same slide pre-treatment as for immunohistochemistry. Primary antibodies included CD3 (as above), CD8 (as above), anti-IgA (rabbit anti-IgA, 1; 10,000, Jackson ImmunoResearch Europe Ltd., Cambridgeshire, UK) and anti-TG2 (CUB7402, 1, 100, Thermo Fisher Scientific). After primary antibody incubation, slides were rinsed and incubated sequentially with immuno-peroxidase polymer as the secondary antibody (Nichirei), CF488 Tyramide (1:100, producing green fluorescence, Biotium, California, USA), secondary antibody peroxidase polymer (Nichirei), and CF568 Tyramide (1:100, producing red fluorescence, Biotium).

**Crypt epithelium proliferation**

A hallmark of gluten-induced mucosal damage is the increased cell proliferation activity in the crypts. We found the best-known immunohistochemical proliferation marker Ki-67 not suitable for quantitation because nearly all crypt epithelium cells were labelled with varying degrees of staining intensity. Instead, we chose another immunohistochemical cell cycle-specific marker, Cyclin B1, which labels cells in the late G2 and M phases of the cell cycle. The fraction of proliferating crypt cells was measured on slides stained immunohistochemically for Cyclin B1, and the proliferating fraction was defined as the percentage of Cyclin B1-positive cells over the total number of crypt cells. In the gluten challenge setting, the percentage of Cyclin B1-positive crypt epithelium cells increased significantly from a mean of 22.87% of crypt cells (range 10-29) to 32.87% of crypt cells (p<0.001; range 14-56). Cyclin B1 values correlated well with crypt depth values (r=0.63, p<0.001), as expected.

**RNA extraction from biopsy samples**

For RNA extraction, ~50 tissue sections (thickness 3-4 um) were cut from paraffin blocks and collected in test tubes. RNA from PAXgene-fixed blocks was extracted using the PAXgene Tissue RNA kit (Qiagen # 765134, Venlo, Netherlands) using an automated robotic nucleic acid extraction system (Qiagen QIAcube, #9001885). In formalin-fixed paraffin-embedded (FFPE) blocks the extraction was done according to the manufacturer's (QIAGEN) protocol for FFPE blocks using the same kit. RNA concentrations were determined with a NanoDrop spectrophotometer (Thermo Fisher Scientific), and RNA quality was determined with a Fragment Analyser (Advanced Analytical, Iowa, USA) with a Standard Sensitivity RNA Analysis Kit (#DNF-471-0500, Advanced Analytical).

**Quantitative real-time PCR using RT^2^ Profiler PCR Arrays**

Genomic DNA was eliminated, and cDNA was synthesized by using the RT^2^First Strand Kit according to the protocol provided by the manufacturer (Qiagen #330401). cDNA was synthesized in quadruplicates of 300 ng of RNA per sample, after which cDNA was mixed with RT^2^ SYBR Green Mastermix (Qiagen #330509) and loaded into a 384-well array. Each sample was loaded in quadruplicate on one array plate and run on a Bio-Rad CFX384^TM^ real-time cycler with the cycling conditions recommended by the array manufacturer (Qiagen, #330231 PAHS-011ZA).

**RNASeq**

The RNA integrity level was measured for each RNA sample using the TapeStation 4200 (Agilent, California, USA) to obtain an RNA integrity number (RINe). The library preparation was done using the QIAseq UPX 3’ Transcriptome Kit (Qiagen). A total of 10 ng purified RNA was converted into cDNA NGS libraries. During reverse transcription, each cell was tagged with a unique ID (up to 384 different IDs), and each RNA molecule was tagged with a unique molecular index (UMI). Then, RNA was converted to cDNA. The cDNA was amplified using PCR, and during the PCR, indices were added. Next, the PCR the samples were purified. Library preparation QC was performed using TapeStation 4200 (Agilent) based on the quality of the inserts. The concentration measurements of the libraries were used to pool the libraries in equimolar ratios. The library pools were quantified using qPCR. The library pools were then sequenced on a NextSeq500 sequencing instrument (Illumina Inc, California, USA) according to the manufacturer’s instructions. Raw data were de-multiplexed, and FASTQ files for each sample were generated using bcl2fastq software (Illumina Inc.). FASTQ data were checked using the FastQC tool. Data were analysed using the GeneGlobe bioinformatics tool (Qiagen). UMI was normalised using DESeq2 (Bioconductor, www.bioconductor.org).

**Data analysis**

Data were analysed with RT2 Profiler PCR Array Data Analysis v. 3.5. (http://pcrdataanalysis.sabiosciences.com/pcr/arrayanalysis.php). For each patient sample, four measurements were taken before the challenge and four measurements after the challenge. These four measurements were grouped, and the data quality was checked. Each group of four measurements passed the PCR Array reproducibility, RT efficiency and Genomic DNA contamination tests. Gene expression data were normalised to the arithmetic mean of the expression of the housekeeping genes ACTB, B2M, GAPDH, HPRT1 and RPLP0.
