# Supplementary material for: Histological, immunohistochemical and mRNA gene expression responses in coeliac disease patients challenged with gluten using PAXgene fixed paraffin-embedded duodenal biopsies
Source: BMC Gastroenterol. 2019 Nov 15;19:189. doi: 10.1186/s12876-019-1089-7 (PMC6858741; doi:10.1186/s12876-019-1089-7)
Supplement: Supplementary file 2 — Additional file 2: Table S1. Conversion of villous height:crypt depth ratio and CD3+ intraepithelial lymphocyte density results of PAXgene-fixed duodenal biopsies to Marsh class and vice versa. [file 12876_2019_1089_MOESM2_ESM.docx]

| **Table S1.** Conversion of villous height:crypt depth ratio and CD3^+^ intraepithelial lymphocyte density results of PAXgene-fixed duodenal biopsies to Marsh class and vice versa. | | |
| --- | --- | --- |
| Marsh class | Villous height:crypt depth ratio^†^ | IELs (CD3^+^; per 100 enterocytes)^‡^ |
| M0 | ≥2.3 | <25 |
| M1 | ≥2.3 | ≥25 |
| M2 | 1.8 - 2.3 | Any |
| M3a | 1.1 - 1.7 | Any |
| M3b | 0.5 - 1.0 | Any |
| M3c | 0.0-0.4 | Any |
| † Determined from H&E-stained digital whole-slide images using Celiac Slide Viewer. | | |
| ‡ Determined from CD3-stained digital whole-slide images using AutoIEL software. | | |
