# Supplementary figures and images for: Histological, immunohistochemical and mRNA gene expression responses in coeliac disease patients challenged with gluten using PAXgene fixed paraffin-embedded duodenal biopsies
Source: BMC Gastroenterol. 2019 Nov 15;19:189. doi: 10.1186/s12876-019-1089-7 (PMC6858741; doi:10.1186/s12876-019-1089-7)

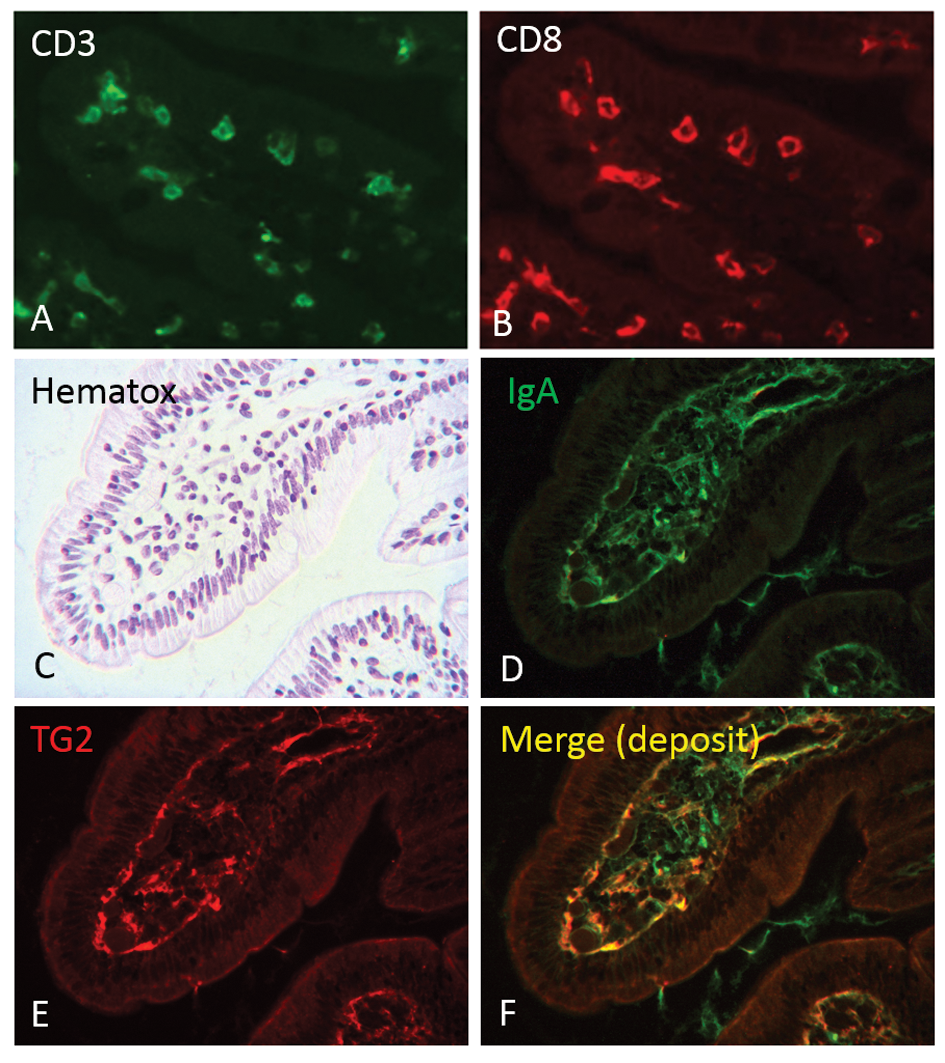

Supplement: Supplementary file 3 — Additional file 3: Figure S1. Immunohistochemical stainings in PAXgene fixed specimens. CD3 (A) and CD8 staining (B) of PAXgene-fixed specimens for analysis of CD3+CD8− lymphocytes in the diagnosis of refractory coeliac disease. Small intestine mucosal immunoglobulin (Ig) A deposits in duodenal specimens in a coeliac disease patient are also shown. First was normal haematoxylin and eosin staining (C), followed by IgA staining (green (D)) and transglutaminase 2 (TG2) (red (E)). The subepithelial colocalization of IgA and TG2 can be seen in yellow (F). [file 12876_2019_1089_MOESM3_ESM.tif]

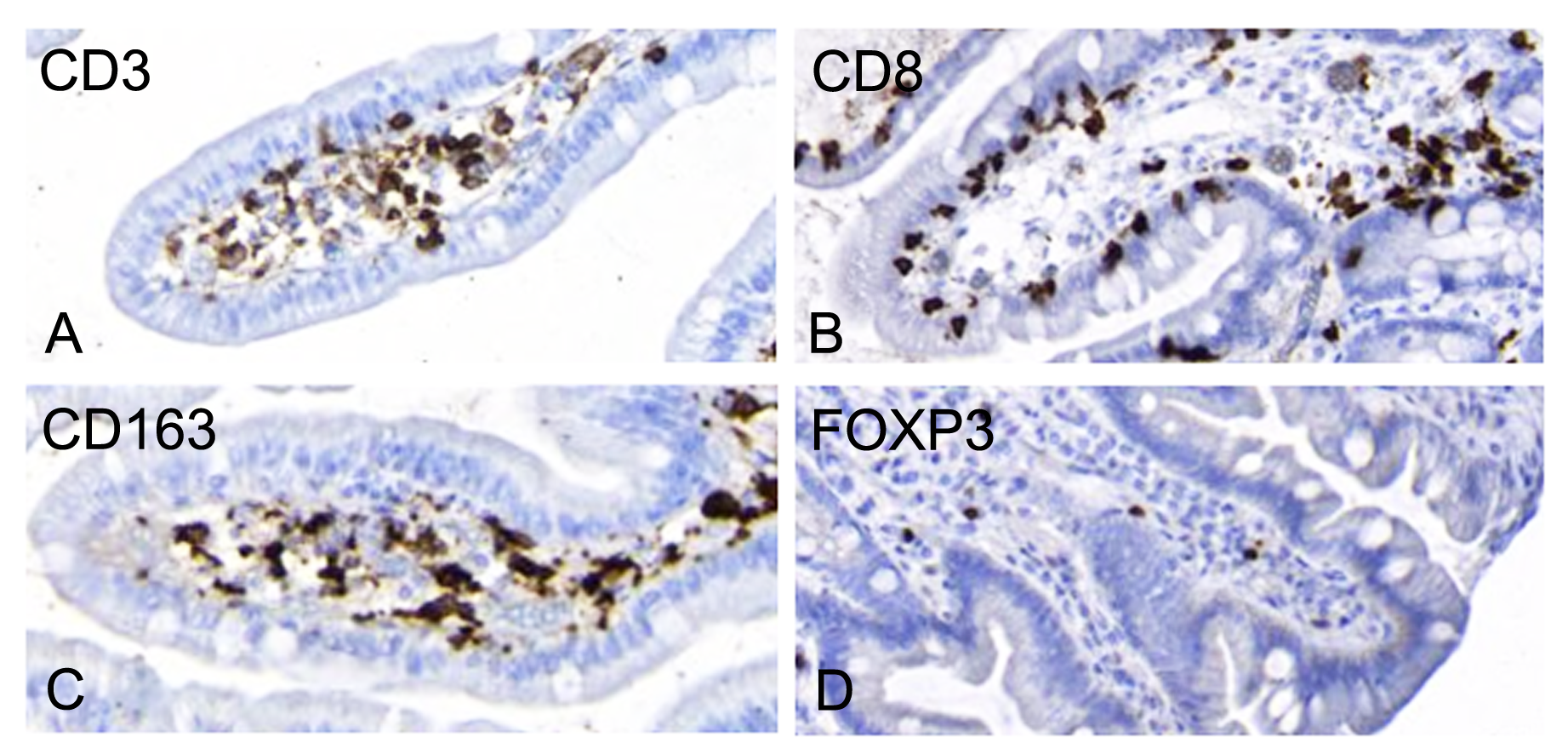

Supplement: Supplementary file 4 — Additional file 4: Figure S2. CD4- (A), CD8- (B), CD163- (C) and FOXP3-stained (D) lymphocytes in PAXgene-fixed specimens. [file 12876_2019_1089_MOESM4_ESM.tif]
